# Differences in Velopharyngeal Structure during Speech among Asians Revealed by 3-Tesla Magnetic Resonance Imaging Movie Mode

**DOI:** 10.1155/2015/126264

**Published:** 2015-07-26

**Authors:** Kulthida Nunthayanon, Ei-ichi Honda, Kazuo Shimazaki, Hiroko Ohmori, Maristela Sayuri Inoue-Arai, Tohru Kurabayashi, Takashi Ono

**Affiliations:** ^1^Orthodontic Science, Graduate School, Tokyo Medical and Dental University, 1-5-45 Yushima, Bunkyo-ku, Tokyo 113-8549, Japan; ^2^Faculty of Dentistry, Naresuan University, Phitsanulok 65000, Thailand; ^3^Oral and Maxillofacial Radiology, Graduate School, University of Tokushima, 3-18-15 Kuramoto-cho, Tokushima 770-8504, Japan; ^4^Oral and Maxillofacial Radiology, Graduate School, Tokyo Medical and Dental University, 1-5-45 Yushima, Bunkyo-ku, Tokyo 113-8549, Japan; ^5^Maxillofacial Orthognathics, Graduate School, Tokyo Medical and Dental University, 1-5-45 Yushima, Bunkyo-ku, Tokyo 113-8549, Japan

## Abstract

*Objective.* Different bony structures can affect the function of the velopharyngeal muscles. Asian populations differ morphologically, including the morphologies of their bony structures. The purpose of this study was to compare the velopharyngeal structures during speech in two Asian populations: Japanese and Thai. *Methods.* Ten healthy Japanese and Thai females (five each) were evaluated with a 3-Tesla (3 T) magnetic resonance imaging (MRI) scanner while they produced vowel-consonant-vowel syllable (/asa/). A gradient-echo sequence, fast low-angle shot with segmented cine and parallel imaging technique was used to obtain sagittal images of the velopharyngeal structures. *Results.* MRI was carried out in real time during speech production, allowing investigations of the time-to-time changes in the velopharyngeal structures. Thai subjects had a significantly longer hard palate and produced shorter consonant than Japanese subjects. The velum of the Thai participants showed significant thickening during consonant production and their retroglossal space was significantly wider at rest, whereas the dimensional change during task performance was similar in the two populations. *Conclusions.* The 3 T MRI movie method can be used to investigate velopharyngeal function and diagnose velopharyngeal insufficiency. The racial differences may include differences in skeletal patterns and soft-tissue morphology that result in functional differences for the affected structures.

## 1. Introduction

Two potential complications after primary surgery for cleft palate repair are oronasal fistula and persistent velopharyngeal insufficiency (VPI) [1–4]. In VPI, inadequate soft palate function results in its inability to lift and thus produce a complete seal with the posterior pharyngeal wall. This condition affects normal speech production and typically manifests as hypernasality and the nasalization of oral sounds [5]. The levator veli palatini (LVP) muscle is the primary muscle for velar elevation [6]. Its origins are the cartilaginous portion of the auditory tube and the petrous portion of the temporal bone. The LVP runs parallel to the Eustachian tube and inserts at the palatine aponeurosis and the midsection of the velum [7–9]. Cadaver and histological studies have shown that 40% of muscle length in the velum is contributed by the LVP [9, 10]. In addition to being part of the velum, the LVP acts as its muscle sling [10]. Contraction of the LVP influences the superior and posterior movements of the velum from the latter's midpoint, which provides the seal between the nasopharynx and oropharynx during speech production [11–15]. Other muscles, such as the palatoglossus and palatopharyngeus muscles, also assist in normal velopharyngeal closure [16].

VPI also occurs in submucous cleft palate and as a postsurgical complication of adenoidectomy [17]. Nasal endoscopy and fluoroscopy are commonly used in the diagnosis of VPI. However, nasal endoscopy requires a high degree of tolerance by the patient, whereas fluoroscopy exposes the patient to radiation [17–19]. The role of magnetic resonance imaging (MRI) in evaluating VPI has been the focus of several studies [20–23]. Among the advantages of MRI are its noninvasiveness, its ability to clearly show the anatomy, and its reproducibility [20–23]. Thus, MRI has been used to investigate velopharyngeal structure and function [6, 14, 22–24], with several researchers using dynamic MRI to evaluate velopharyngeal function in real time [14, 25–27].

All of the muscles of the soft palate have bony origins. For example, the LVP originates from the temporal bone, the musculus uvulae from the palatine bone, and the tensor palatine from the sphenoid bone [7]. Bony structures are known to vary depending on age, race, and sex [28–34]. Racial differences have been demonstrated using lateral cephalometric analysis. For example, using lateral cephalometric radiography, Beugre et al. [28] found differences in the dental, skeletal, and soft-tissue facial morphologies of three African populations. de Freitas et al. [33, 34] demonstrated variations in the bony structures of Brazilian populations: white Brazilians have a larger upper anterior facial height whereas black Brazilians have a larger lower facial height. Moreover, black, but not white, Brazilians have a bimaxillary skeletal, dentoalveolar, and soft-tissue protrusion, although both groups have normal occlusion. Furthermore, there is greater protrusion of the upper and lower lips in black than in white Brazilians, but their lip thicknesses are similar. Asian populations have characteristic skeletal and soft-tissue morphologies that are different from those of Caucasian populations [35–42]. Somboonsap et al. (2011) used lateral cephalometric radiographs to characterize the morphology of the hard tissues of patients with obstructive sleep apnea syndrome and compared the cephalometric data of the study's Caucasian, Hispanic, African American, Singaporean, Japanese, and Thai participants. The authors reported that East Asians have more prominent upper and lower jaws than Westerners.

Differences in the bony structures that serve as the origins of the muscles of the soft palate together with differences in soft-tissue morphology might account for the observed differences in velopharyngeal function. For example, MRI studies have shown that males have a larger LVP than females [15, 24, 43]. However, there are few MRI studies on racial differences. Thus, it may be that the morphological differences among Asian populations include different velopharyngeal structures. The aim of this study was to use dynamic MRI to investigate the moment-to-moment changes in velopharyngeal structures during speech production in Asian populations, specifically, between Japanese and Thai subjects.

## 2. Methods

### 2.1. Subjects

Since gender is known to have effects on velopharyngeal structures [15, 43], only female subjects were included in this study. Ten healthy adult females (five Japanese and five Thai) participated in this study. All subjects had a normal body mass index (BMI), thus avoiding variations in the pharyngeal airways because of obesity. The mean age of the Japanese group was 28.2 ± 1.3 (mean ± standard deviation (SD)) years and that of the Thai group 29.2 ± 1.3 years. The two groups did not significantly differ with respect to age, height, weight, and BMI (Table 1). None of the participants had a history of neurological, craniofacial, musculoskeletal, speech, or hearing disorders, nor did they have any sign of cold, allergic rhinitis, or respiratory infectious disease at the time of the experiment. Written informed consent was obtained from all ten subjects prior to their participation in the study. The research protocol was approved by the Institutional Ethical Review Board of the Tokyo Medical and Dental University (number 886).

### 2.2. Magnetic Resonance Imaging

Custom-made circuitry was connected to a 3-Tesla (3 T) MRI apparatus (Magnetom Spectra, Siemens, Germany), which included a head and neck coil. At the start of the experiment, the subject was placed in the supine position, with her head stabilized inside the head coil, and fitted with headphones, which provided an auditory cue to synchronize pronunciation with the scanning time. A programmed external trigger pulse was used to control the timing scan sequence and to provide the acoustic cue, allowing the scan to be synchronized with pronunciation. A fiber-optic microphone (FOMRI, Phone-or, Or-Yehuda, Israel) was placed in front of the subject's lips to capture her pronunciation. A connected digital recorder (PMD670, 2-channel solid state recorder, Marantz Professional, Kingsbridge House, Middlesex, UK) was used to record the pronunciation and the external trigger pulse. Image acquisition consisted of a gradient-echo sequence, fast low-angle shot (FLASH) with segmented cine and parallel imaging technique (GRAPPA). The mid-sagittal plane was imaged using the following settings: repetition time (TR) = 22.5 ms, echo time (TE) = 2.07 ms, flip angle (FA) = 12°, field of view = 256 × 256 mm, matrix size = 256 × 128, pixel size = 1 × 2 mm, slice thickness = 4 mm, and acceleration factor = 2.

### 2.3. Speech Task

An external trigger pulse was fed to the MRI scanner 16 times and the subjects were required to repeat (echo) the vowel-consonant-vowel (VCV) syllable (/asa/) 16 times at 1500 ms intervals and in a synchronized manner in response to the auditory cue. The consonant /s/ was chosen because a previous study showed that during /s/ production the velopharyngeal structures are not affected by the supine position [44]. The subjects were asked to breathe between repetitions of the VCV articulation and to return the tongue and mandible to the resting position at the beginning and end of every pronunciation.

### 2.4. Sound Data Manipulation

**Figure 1:**
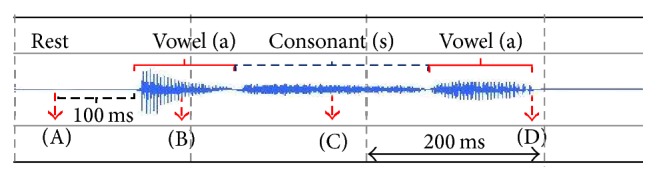
A representative sound wave and determination of the timing used in the analysis. Images acquired during the rest stage, 100 ms before production of the first vowel (A stage), in the middle of production of the first vowel (B stage) and the consonant (C stage), and immediately after production of the second vowel (D stage) were analyzed.

The recorded sound data were manipulated using the free software SoundEngine (Code Helium, Japan), which allows the time of articulation and the sound wave to be recorded. The manipulated sound data also assisted in stage determination. Images acquired during the rest phase (before pronunciation, 100 ms), the first vowel (the middle of the first vowel /a/), the consonant (the middle of consonant /s/), and after speech (the end of the second vowel /a/) were chosen for analysis (Figure 1). The speech duration of each subject was measured and compared between the Japanese and Thai subjects. The start and finish points of each pronunciation were determined when the sound signal exceeded 3 SD of the baseline value.

### 2.5. Area of Interest and Measurements

**Figure 2:**
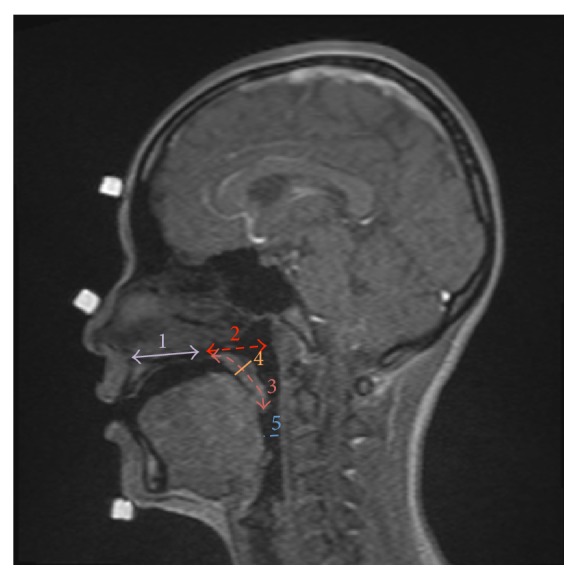
The measurement parameters: 1, hard-palate length; 2, velopharyngeal depth; 3, velar length; 4, velar thickness; 5, retroglossal space. See the text for definitions.

Four images, from the four above-described stages, were obtained at five distances from each subject (Figure 2). The measurement parameters were adapted from the study of Perry [44]. All parameters and their definitions are shown in Table 2.

### 2.6. Statistical Analysis

Each image was measured five times over 5 days. A single examiner (KN) conducted the measurements to avoid interobserver error. Intraobserver reliability was assessed by intraclass correlation coefficients (ICC). The measurement errors determined by the ICC were very small (range: 0.95–0.99), showing that the measurements were reproducible. Levene's test (*F*) was used to assess the equality of variances; ANOVA and Tukey's test were used to compare the parameters of the Japanese versus Thai subjects with respect to the different stages of pronunciation. The duration of speech in the two groups was compared by first using the Kolmogorov-Smirnov *Z* test to verify the suitability of a paired sample *t*-test. Significance was defined as *p* < 0.05.

## 3. Results

### 3.1. Japanese Subjects

**Figure 3:**
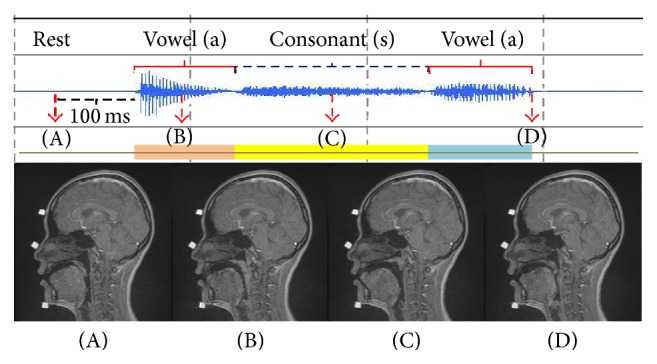
Representative sound data combined with movie MRI of a Japanese subject. A–D are the corresponding sound signals and MR images.

Four stages were analyzed in this study: rest stage, 100 ms before production of the first vowel (A stage), in the middle of production of the first vowel (B stage) and the consonant (C stage), and immediately after production of the second vowel (D stage). Representative sound data combined with the movie MRI of a Japanese subject are shown in Figure 3. At the A stage, the articulators were in the rest position, with the velum placed on the posterior aspect of the tongue. At the B stage, the velum was elevated in a superoposterior direction and made contact with the posterior pharyngeal wall. In only one Japanese subject was there no contact between the velum and the posterior pharyngeal wall during this stage; instead, the tongue moved in an anterior direction. The retroglossal space was diminished during the B stage compared with the rest stage. At the C stage, the tongue actively moved in the anterior direction towards the premaxillary area. The velum achieved a higher position than during vowel pronunciation and the retroglossal space was enlarged. At the D stage, the tongue moved backward and the velum downward such that the retroglossal space again became smaller.

### 3.2. Thai Subjects

**Figure 4:**
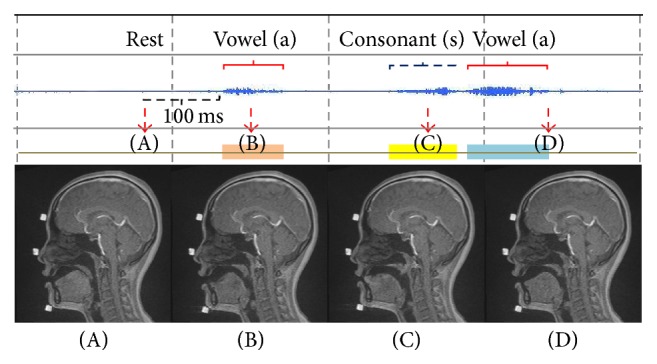
Representative sound data combined with movie MRI of a Thai subject. A–D are the corresponding sound signals and MR images.

Figure 4 shows representative data from the Thai group. Although two groups pronounced the same VCV syllable (/asa/), Thai subjects trended to produce a significantly shorter (*p* < 0.05) consonant than Japanese subjects (Table 3). In the Thai group, there was a short break between the first vowel and the consonant. During the A stage, the velum of Thai subjects was placed on the posterior tongue, as observed in the Japanese group. During the B stage, however, the Thai group differed from the Japanese group in that an obvious groove formed near the tip of the tongue. The velum was elevated but there was no contact between it and the posterior pharyngeal wall. In only one subject was there contact between the two structures during this stage. During the C stage, the tongue moved anteriorly and the velum was further elevated, similar to the Japanese group. Contact between the velum and the posterior pharyngeal wall was observed. Finally, in the D stage, the tongue moved posteriorly and the velum moved downward.

### 3.3. Differences in the Velopharyngeal Structures between the Two Groups

**Figure 5:**
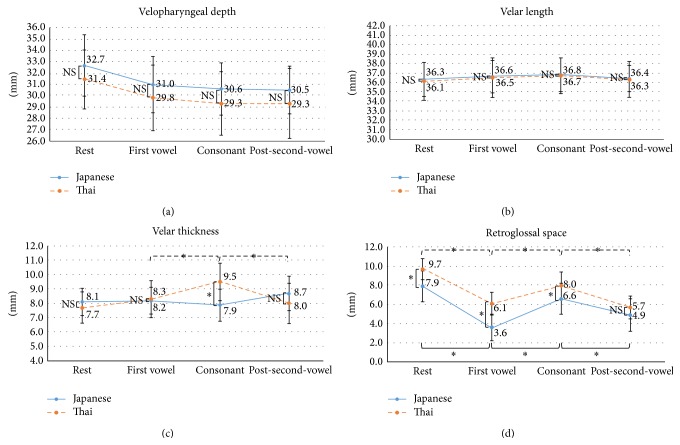
Time-to-time changes in the velopharyngeal structures of Japanese and Thai subjects. The graphs show the dimensional changes in each area of interest: velopharyngeal depth (a), velar length (b), velar thickness (c), and retroglossal space (d). The *x*-axis shows the measurement stages and the *y*-axis shows the distance in millimeters. ^∗^ *p* < 0.05; NS: not significant.

A time-to-time quantitative comparison of the data of the Japanese and Thai groups is shown in Figure 5. Thai subjects had a significantly longer hard palate than Japanese subjects (36.36 ± 2.22 and 33.66 ± 1.82, resp.) but the velopharyngeal depth did not differ significantly between the two groups at any stage (Figure 5(a)). The temporal changes were the same: the velopharyngeal depth was deepest during the rest stage and decreased during pronunciation. A combined assessment of the length of the hard palate and the velopharyngeal depth showed a longer dimension in the Thai group than in the Japanese group at every stage: rest stage (67.79 ± 2.66 versus 66.32 ± 3.85), first vowel stage (66.25 ± 2.43 versus 64.41 ± 3.37), consonant stage (65.58 ± 1.70 versus 64.15 ± 3.17), and the last vowel stage (65.89 ± 2.05 versus 66.04 ± 3.42). Velar length did not differ significantly between the two groups and was stable at every stage of measurement (Figure 5(b)). The velar thickness was similar at the rest, first vowel, and post-second-vowel stages; however, during the consonant stage the velum became significantly thicker in the Thai than in the Japanese subjects. In the latter, velar thickness was nearly stable at every measurement stage whereas in the Thai group the velum became markedly thicker during production of the consonant than during production of the first vowel but it significantly decreased in thickness after production of the second vowel (Figure 5(c)). The retroglossal space of the Thai group was significantly wider than that of the Japanese group during the rest, first vowel, and consonant stages. There was no significant difference between the two groups in the post-second-vowel stage, and similar patterns in the dimensional change of the retroglossal space were observed; that is, the space was broad in the rest stage, significantly narrower during the first vowel, markedly wider during consonant production, and narrower again during the post-second-vowel stage (Figure 5(d)).

### 3.4. Speech Duration in the Japanese and Thai Groups

**Figure 6:**
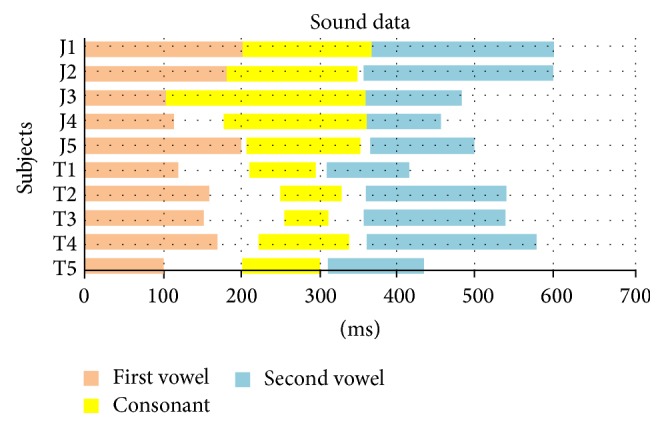
Speech duration in the 10 Japanese (J) and Thai (T) subjects. The *x*-axis shows the duration of speech in ms.

The speech duration of all subjects is shown in Figure 6 and the mean speech duration of the two groups in Table 3. Japanese subjects had a tendency to produce sounds continuously whereas Thai subjects paused at the end of every sound; the pause between the first vowel and the consonant was longer than that between the consonant and the second vowel. Thai subjects produced significantly shorter consonants than Japanese subjects, while there was no difference in the vowel sounds of the two groups.

## 4. Discussion

MRI movie mode is an effective and noninvasive method of studying velopharyngeal function and articulation in real time [25, 27]. Although subjects are in the supine position during the experiments, gravity has been shown to only minimally affect velar and pharyngeal dimensions [44]. In fact, for the complete production of normal sound, adequate function of the soft palate is needed for the formation of an adequate seal with the posterior pharyngeal wall and for separating the nasopharynx from the oropharynx, to prevent both nasal escape during pressure consonants and hypernasality [5]. MRI movie mode can also be used to visualize the events that form the basis of the diagnosis, treatment, and prognosis of VPI.

Japanese and Thai groups did not differ significantly with respect to velopharyngeal depth whereas Thai subjects had a significantly longer hard palate. These findings are in agreement with previous studies in which lateral cephalometric analysis showed a normal skeletal pattern in Japanese and Thai populations [41, 42]. Our comparison of cephalometric norms between Japanese and Thai females [41, 42] showed some differences: the Thai subjects had larger sella–nasion–A and sella–nasion–B point angles (SNA and SNB) and facial angles than their Japanese counterparts, while the A point–nasion–B point angles (ANB) were similar (Thai: SNA = 85.22 ± 3.94, SNB = 81.26 ± 3.68, and ANB = 3.96 ± 1.70; Japanese: SNA = 82.32 ± 3.45, SNB = 78.90 ± 3.45, and ANB = 3.39 ± 1.77) [41, 42]. Thai females had a more protruding profile than Japanese females, as indicated by the facial angle (Thai = 90.36 ± 2.59, Japanese = 84.83 ± 3.05) and the angle of convexity (Thai = 9.42 ± 4.76, Japanese = 7.58 ± 4.95) [41, 42]. This suggests that the size difference in the upper airway, especially at the site of the velopharyngeal constriction during pronunciation, is negligible despite the significant differences between the Thai and the Japanese regarding the hard tissues of the oropharyngeal region.

The velar lengths of the two groups were similar at every stage of measurement, consistent with the results of a previous study [44] that used MRI to evaluate the velopharyngeal structures in white females placed in the upright and supine positions. In our study, the mean velar lengths during rest (Japanese: 36.3 mm, Thai: 36.1 mm) and consonant production (Japanese: 36.8 mm, Thai: 36.7 mm) were shorter than the values reported by Perry [44] (40.3 mm during rest and 43.9 mm during consonant /s/ production). The difference may be attributable to the racial differences in the morphology of the craniofacial region: brachycephalic in the Mongoloid population and dolichocephalic in the Caucasian population.

The mean velar thickness in the Asian females in this study was smaller than that reported for the white females in the Perry's study [44]: during the resting stage, 8.1 mm in Japanese, 7.7 mm in Thai, and 9.7 mm in whites; during the consonant stage: 7.9 mm in Japanese, 9.5 mm in Thai, and 11.1 mm in whites. While both studies showed velar thickening during consonant production, our Japanese and Thai subjects differed in that in the latter group thickening of the velum during consonant production was significantly greater. This difference may affect the quality of consonant sound because although the participants were instructed to produce the same consonant, the consonant sounds of the two groups of subjects were unique, with the Thai group producing a shorter consonant sound than the Japanese group (Figure 6). This difference may be related to the amount of velar thickening needed to clearly produce a consonant sound and thus to compensate for the large retroglossal space within a short time frame.

The mean retroglossal space of Asians seems to be smaller than that of white females, based on a comparison between our findings and those of Perry [44] (rest stage: 7.9 mm in Japanese, 9.7 mm in Thai, and 10.8 mm in whites; consonant stage: 6.6 mm in Japanese, 8.0 mm in Thai, and 15.3 mm in whites). The reason why the retroglossal space of Asians decreases in size from the rest stage to consonant production while in whites it increases is unclear.

According to our findings, in Japanese and Thai populations velopharyngeal function is nearly the same, except for the difference in velar thickening during consonant production and in the retroglossal space during rest and speech production (Figure 5). Based on our measurements of hard-palate length and on the cephalometric norms reported in previous studies [41, 42], our results suggest that the differences in the soft-tissue morphology among Asian populations reflect differences in their skeletal patterns. Further studies of the interaction between the hard and soft tissues in different races and of the effects of race on velopharyngeal structure and function would certainly be of broad interest but will require much larger sample sizes than used in this study.

## 5. Conclusions

To produce a normal consonant sound, a complete seal in the posterior pharyngeal space is necessary, which requires the participation of the soft palate and related muscles. Our study demonstrated significant differences in hard-palate width, velopharyngeal depth, retroglossal space, and velar thickness during the production of the test consonant by Japanese and Thai subjects. The racial features of the two groups included structural and functional differences in skeletal patterns and soft-tissue morphology.

Using 3 T MRI in movie mode, velopharyngeal structures and their variations in different populations can be precisely observed in real time, thereby improving our understanding of velopharyngeal function. This imaging approach is also useful for the diagnosis and treatment of patients with VPI and in the evaluation of outcome. Studies such as ours will allow patient-tailored treatment of VPI based on gender, race, skeletal patterns, and velopharyngeal structures.

## Figures and Tables

**Table 1:** Demographic data of the two groups.

	Japanese subjects (*N* = 5)	Thai subjects (*N* = 5)	Significance
Age (years)	28.2 ± 1.3	29.2 ± 1.3	NS
Weight (kg)	49.5 ± 5.9	53.2 ± 5.1	NS
Height (cm)	158.3 ± 6.4	161.4 ± 2.3	NS
Body mass index (kg/m^2^)	19.7 ± 0.8	20.4 ± 1.6	NS

NS: not significant.

**Table 2:** The study parameters and their definitions.

Parameter	Definition
Hard-palate length	Distance between the anterior nasal spine (ANS) and the posterior nasal spine (PNS)
Velopharyngeal depth	Distance between the PNS and the posterior pharyngeal wall on the plane parallel to the hard palate
Velar length	Distance between the PNS and the tip of velum on the curvature along the velum
Velar thickness	Distance between the velar knee and velar dimple
Retroglossal space	Distance between the tongue and posterior pharyngeal wall on the plane parallel to the hard palate and through the anteroinferior border of the second vertebra (C2)

**Table 3:** The duration of speech in the Japanese and Thai groups.

Sounds	Japanese (*N* = 5) (ms)	Thai (*N* = 5) (ms)	Significance
First vowel	160.4 ± 47.7	140.4 ± 29.3	NS
Consonant	183.8 ± 42.5	87.0 ± 22.6	∗
Second vowel	164.8 ± 67.5	160.6 ± 46.3	NS

^∗^ *p* < 0.05; NS: not significant.
